# MINDFULNESS AND DEPRESSION ASSOCIATED WITH FALL RISK IN LOW-INCOME COMMUNITY-DWELLING OLDER ADULTS

**DOI:** 10.1093/geroni/igad104.3471

**Published:** 2023-12-21

**Authors:** Aleeyah Uddeen, Renata Komalasari, Ladda Thiamwong

**Affiliations:** University of Central Florida, Orlando, Florida, United States; Penn State Nursing, State College, Pennsylvania, United States; University of Central Florida, Orlando, Florida, United States

## Abstract

Mindfulness is the state of being aware and present using mind-body exercises. Practicing mindfulness and reducing depression can improve balance and reduce fall risk among older adults. The purpose of the study was to examine the correlation between mindfulness and depression with fall risk among community-dwelling older adults in low-income settings. A descriptive cross-sectional survey design was used to assess 103 older adults (males=16, females=87, mean age=75.70, 95% CI±7.19) using questionnaires to measure mindfulness, depression, and fall risk. Mindfulness was measured using the Day-to-Day Experiences, which is a fifteen-item scale with lower scores indicating less awareness. Depression was assessed using the Patient Health Questionnaire-9 (PHQ-9). Fall risk was assessed using the CDC’s Stay Independent checklist. Data analysis was run using SPSS version 28. Spearman rank analysis showed that mindfulness was negatively correlated with fall risk (r=-.437, p<.001), whilst depression was positively correlated with fall risk (r=.486, p<.001). Using regression analysis, we examined if mindfulness and depression predicted fall risk. The results of the multiple regression indicated that the model explained 23% of the variance (R²=.230, F (2,100)=14.97, p<.001). Mindfulness (β=-.060, p=.010) and depression (β=.341, p=.002), respectively, were significantly associated with fall risk. It can be concluded that mindfulness and depression are predictors of fall risk in this study. Older adults, particularly in low-income settings, could potentially benefit from improving mindfulness through mind-body exercises that enhance awareness and mindful movement, as well as from improving mental health, thereby reducing fall risk.

